# Stereoselective Blockage of Quinidine and Quinine in the hERG Channel and the Effect of Their Rescue Potency on Drug-Induced hERG Trafficking Defect

**DOI:** 10.3390/ijms17101648

**Published:** 2016-09-28

**Authors:** Meng Yan, Pan Fan, Yanhui Shi, Lifang Feng, Junnan Wang, Ge Zhan, Baoxin Li

**Affiliations:** 1 Department of Pharmacology, Harbin Medical University, No. 194 Xuefu Road, Nangang District, Harbin 150081, China; yanmeng1214@163.com (M.Y.); shiyanhuihao123@163.com (Y.S.); fmlff123@163.com (L.F.); wangjunnan0611@163.com (J.W.); zhange0403@163.com (G.Z.); 2 Department of Ophthalmology, the Second Affiliated Hospital, Harbin Medical University, No. 148 Baojian Road, Nangang District, Harbin 150081, China; di20071214@163.com

**Keywords:** quinidine, quinine, hERG (human *ether-a-go-go*-related gene), stereoselectivity, pharmacological rescue

## Abstract

Diastereoisomers of quinidine and quinine are used to treat arrhythmia and malaria, respectively. It has been reported that both drugs block the hERG (human *ether-a-go-go*-related gene) potassium channel which is essential for myocardium repolarization. Abnormality of repolarization increases risk of arrhythmia. The aim of our research is to study and compare the impacts of quinidine and quinine on hERG. Results show that both drugs block the hERG channel, with quinine 14-fold less potent than quinidine. In addition, they presented distinct impacts on channel dynamics. The results imply their stereospecific block effect on the hERG channel. However, F656C-hERG reversed this stereoselectivity. The mutation decreases affinity of the two drugs with hERG, and quinine was more potent than quinidine in F656C-hERG blockage. These data suggest that F656 residue contributes to the stereoselective pocket for quinidine and quinine. Further study demonstrates that both drugs do not change hERG protein levels. In rescue experiments, we found that they exert no reverse effect on pentamidine- or desipramine-induced hERG trafficking defect, although quinidine has been reported to rescue trafficking-deficient pore mutation hERG G601S based on the interaction with F656. Our research demonstrated stereoselective effects of quinidine and quinine on the hERG channel, and this is the first study to explore their reversal potency on drug-induced hERG deficiency.

## 1. Introduction

**Figure 1:**
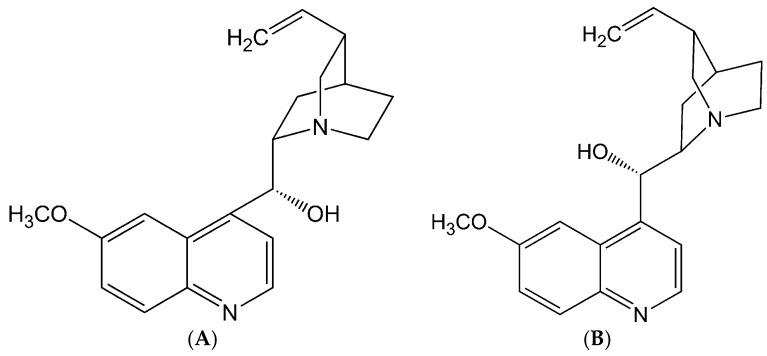
Chemical structure of quinine (**A**) and quinidine (**B**).

Quinidine and its laevorotatory diastereomer quinine (structural formulas are shown in Figure 1) are quinoline alkaloids mainly extracted from cinchona bark [1]. They have similar pharmacological activities in many aspects, but differ from each other in clinical application. Quinidine is widely used in tachyarrhythmia as a class Ia antiarrhythmic agent [2]. Quinine is important for treatment of severe malaria as an antimalarial drug [3]. However, cardiotoxicity is the common serious adverse effect during treatment with both drugs [4,5]. Stereospecific electrophysiologic effects of quinidine and quinine have been reported previously in the heart. In vitro, quinidine prolongs whereas quinine shortens action potential duration recorded in canine cardiac Purkinje fibers [6]. In vivo, quinidine prolongs repolarization time in a canine model of ventricular tachycardia whereas quinine does not [7]. A separate report discovered that quinidine and quinine prolonged the QaT (apex of T wave) interval in a dose-dependent manner measured by the isolated perfused guinea pig heart, with quinine more potent than quinidine [8]. In patients with malaria, quinidine has been reported can cause QT (duration from the start of QRS-wave to the end of T-wave) interval prolongation and torsade de pointes (TdP) [5]. In contrast, quinine induces a slight lengthening of the QTc interval [9]. Drug-induced QT interval prolongation or TdP is frequently associated with the block of hERG (human *ether-a-go-go*-related gene) potassium channel [10]. A previous study reported that quinidine blocked the hERG channel by interaction with Y652 and F656, two critical aromatic residues of drug binding sites in the S6 domain [11]. The aim of our study is to investigate whether stereospecific differences occur in the effect of quinidine and quinine on the hERG channel.

*hERG* encodes a pore-forming subunit of the rapid activating component of the cardiac delayed rectifier potassium current (I_Kr_), loss function of which will lead to delayed repolarization of cardiocytes, presented clinically with prolongation of QT interval and even TdP (a lethal ventricular tachyarrhythmias associated with QT interval prolongation, which could develop into ventricular fibrillation, even sudden cardiac death). A lot of different kinds of medicines prolong the QT interval via hERG channel inhibition. Drug-induced LQTS (long QT syndrome) has become a significant hurdle for the application of therapeutic drugs, as well as in the development of novel drug compounds. All major pharmaceutical companies have to monitor the potential risk of LQTS induced by new or existing drugs [12]. The mechanisms underlying this inhibition are mainly two types: one is blocking the channel directly [13]; another is indirectly decreasing the channel expression on the cell surface, such as disruption of channel forward trafficking to the membrane [14] and promotion of the degradation of channel protein [15,16]. As for the trafficking-defective hERG channel, there were reports that a high-affinity hERG blocker would produce pharmacological rescue. Quinidine, astemizole, cisapride, and E4031 successfully rescued the trafficking-deficient mutation G601S-hERG, and the rescue relied on their interaction with F656 [17]. Terfenadine and fexofenadine reversed N470D-hERG mutation [18]. Astemizole rescued pentamidine and caused hERG trafficking inhibition via competitive interaction with F656 residue [19]. Hence, we hypothesized that the two drugs may produce stereoselective rescue of the drug-triggered hERG trafficking defect based on interaction with F656.

In our study, we used a patch clamp technique to investigate whether quinidine and quinine produce stereoselective blockage in the hERG channel and their dynamics. We also focused on their effects on the expression of channel protein. Finally, we explored whether quinidine and quinine possess reversal efficacy with respect to the drug-induced hERG trafficking defect.

## 2. Results

### 2.1. Stereoselective Difference in hERG Blockage by Quinidine and Quinine

**Figure 2:**
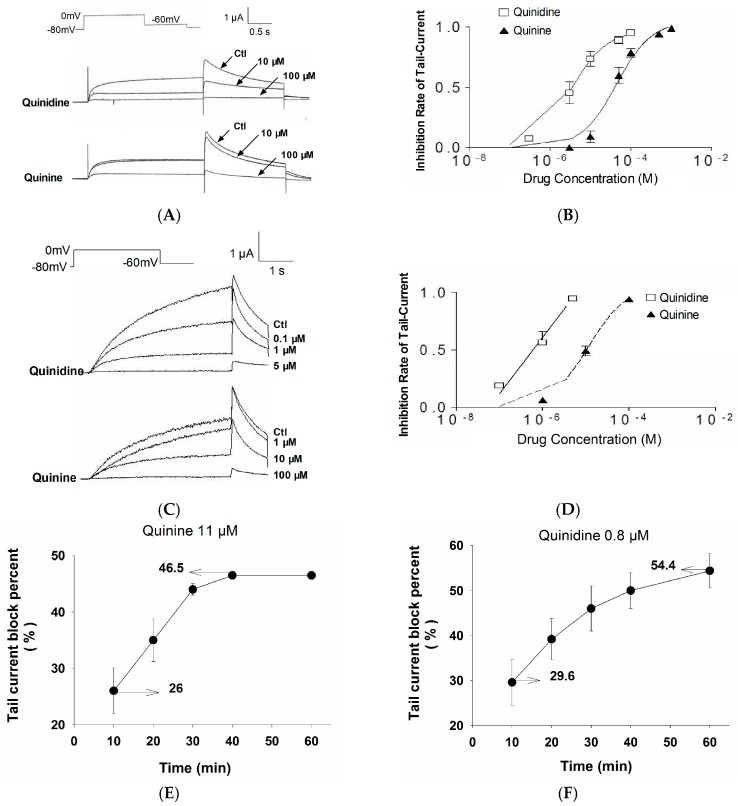
Stereoselective blockage of the hERG channel by quinidine and quinine. (**A**) Protocol and representative currents recorded from *Xenopus* oocytes injected with WT-hERG cRNA. Both quinidine and quinine blocked the hERG current significantly, but quinidine was more potent than quinine at the same concentration; (**B**) The concentration-response curves of blockage of hERG by quinidine and quinine in *Xenopus* oocytes. IC_50_ of quinidine and quinine for WT-hERG was 3.00 ± 0.03 μM and 44.0 ± 0.6 μM, respectively; (**C**) Protocol and representative currents recorded from *Ltk*^−^ cells transfected with WT-hERG cDNA. Results were similar to those found in *Xenopus* oocytes; (**D**) The concentration-response curves of blockage of hERG by quinidine and quinine in *Ltk*^−^ cells. IC_50_ of quinidine and quinine for WT-hERG was 0.8 ± 0.1 μM and 11 ± 3 μM, respectively; (**E**,**F**) Time-dependent blockage of hERG by quinine (**E**) and quinidine (**F**) in *Xenopus* oocytes at the concentrations of their IC_50_ value for *Ltk*^−^ cells. Abbreviations: ctl, control; WT-hERG, wild type hERG.

To find out whether a difference in efficiency on hERG inhibition existed between quinidine and quinine, we recorded hERG currents at different concentrations of quinidine and quinine from *Xenopus* oocytes which were injected with wild type-hERG (WT-hERG) cRNA. Figure 2A illustrates the protocol and representative examples of hERG currents. Quinidine produces more than 50% blockage of hERG current at 10 μM, whereas little blockage was observed at this concentration of quinine. However, the inhibiting effect of quinine was significantly enhanced at a higher concentration (100 μM). Figure 2B shows the concentration-response curves. The horizontal axis represents the concentration of quinidine and quinine; the vertical axis represents the inhibition ratio of the tail currents. The mean IC_50_ (the half maximal inhibitory concentration) was 3.00 ± 0.03 μM of quinidine versus 44.0 ± 0.6 μM of quinine.

To exclude the possibility that stereospecificity existed only when hERG was expressed in *Xenopus* oocytes, we performed a similar experiment in *Ltk^−^* cells which were transfected with WT-hERG cDNA (Figure 2C). The IC_50_ was 0.8 ± 0.1 μM of quinidine and 11 ± 3 μM of quinine in *Ltk^−^* cells (Figure 2D).

The above results showed that the concentration required to block hERG in *Xenopus* oocytes is approximately 4-fold higher than that in *Ltk^−^* cells. However, if we prolonged the perfusion time of quinidine or quinine at the concentration of their IC_50_ measured in *Ltk^−^* cells (0.8 μM and 11 μM, respectively), the hERG currents recorded from *Xenopus* oocytes can be blocked about 50% after 1 h superfusion (Figure 2E,F).

Taken together, quinine was ~14-fold less potent than quinidine in hERG blockage. These data indicate that quinidine and quinine produce stereospecific effects when hERG is expressed in *Xenopus* oocytes or *Ltk^−^* cells. Both quinidine and quinine showed time-dependent blockage of the hERG channel.

### 2.2. Effects of Quinidine and Quinine on Gating Properties of the hERG Channel

**Figure 3:**
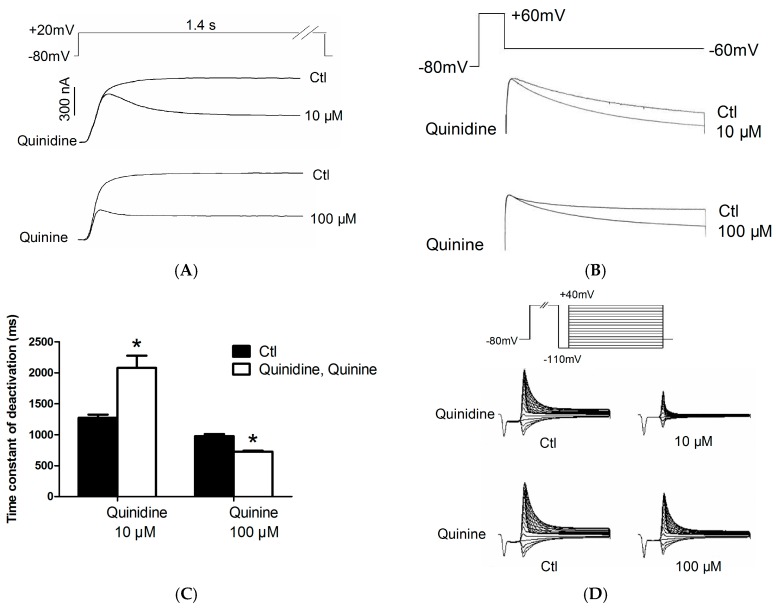

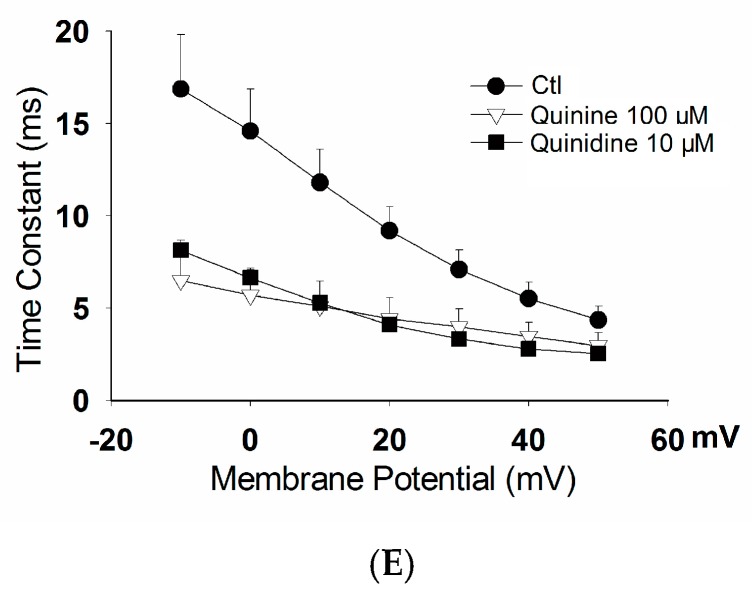
Effects of quinidine and quinine on gating properties of the hERG channel in *Xenopus* oocytes. (**A**) Quinidine and quinine block open channels. The currents were elicited from a holding potential of −80 mV to a depolarizing potential of +20 mV for 1.4 s; (**B**) Protocol and representative currents of deactivation; (**C**) The mean time constants of deactivation before and after drug treatments. Quinidine slowed deactivation whereas quinine accelerated deactivation. * *p* < 0.05, *n* = 10; (**D**) Representative current tracing for inactivation using a three-pulse protocol; (**E**) The time constant for onset of inactivation before and after exposure to 10 μM quinidine or 100 μM quinine. Both quinidine and quinine accelerated the channel inactivation. Abbreviation: ctl, control.

First, we investigated whether the blockage of hERG by quinidine and quinine requires channel activation. The concentration of quinidine and quinine chosen based on their dose-response curves showed similar inhibition levels with respect to hERG blockage, i.e., at a concentration approximately 3-fold higher than their IC_50_ (quinidine at 10 μM was compared to quinine at 100 μM). Figure 3A shows the hERG currents expressed in *Xenopus* oocytes elicited by the protocol seen in the inset. Currents were recorded first under control conditions and then after superfusion with 10 μM quinidine or 100 μM quinine for 10 min. During quinidine treatment, the first pulse of depolarization resulted in a reduction of peak current level and current amplitude and then the current rapidly declined to a lower steady-state level. The rate of decay of current was enhanced by increasing the concentration of quinidine (data not shown). Similarly, quinine at 100 μM also showed an immediate reduction of the peak current followed by a time-dependent decline to a steady state level. These data suggest that both quinidine and quinine block open hERG channels.

A deactivation current was obtained by a repolarizing pulse at −60 mV for 5-s after a 1-s depolarizing pulse of +60 mV from a holding potential of −80 mV (Figure 3B). Figure 3C shows the time constants of deactivation under control conditions and in the presence of 10 μM quinidine or 100 μM quinine. It was prolonged by 10 μM quinidine from 1274.00 ± 49.69 ms to 2079.00 ± 196.80 ms, while shortened from 976.70 ± 32.42 ms to 726.00 ± 13.80 ms by 100 μM quinine. In summary, quinidine slowed deactivation rate constants whereas quinine significantly accelerated them.

To measure the effect of quinidine and quinine on the inset of inactivation, we used a triple pulse protocol (Figure 3D). The channels are first depolarized to positive potential (+40 mV) with a 300-ms pulse to increase the probability of inactivation, and then are transiently hyperpolarized to −110 mV for a short time (25 ms) so that the channels do not have sufficient time to deactivate and nearly all of the channels enter into the open state. Following the prepulse, a series of test pulses are delivered to potentials ranging from −110 to +40 mV to record the inactivating currents. The time constants of inactivation in the absence and presence of 10 μM quinidine or 100 μM quinine are shown in Figure 3E. As shown in the figure, the time constant at +10 mV was shortened from 16.86 ± 2.95 ms to 8.13 ± 0.55 ms for 10 μM quinidine and 6.50 ± 1.48 ms for 100 μM quinine. It signified that quinidine and quinine accelerated the channel inactivation.

### 2.3. F656C Abrogates the Stereospecific Differences between Quinidine and Quinine

**Figure 4:**
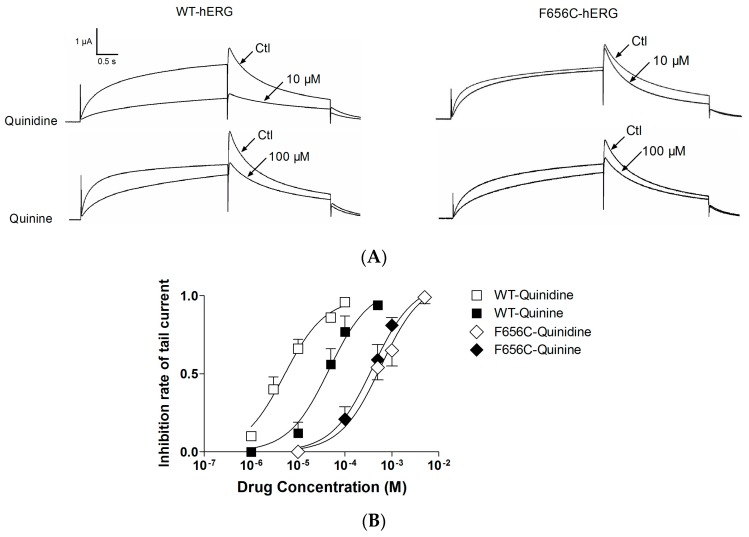
F656C-hERG abrogates the stereospecific differences between quinidine and quinine. (**A**) Representative currents of WT-hERG and F656C-hERG after quinidine or quinine treatment. The protocol is illustrated in Figure 2A. Quinidine 10 μM exhibited no significant blockage and quinine 100 μM exhibited slight blockage of F656C-hERG; (**B**) The extent of blockage of WT-hERG or F656C-hERG by quinidine and quinine. IC_50_ of quinidine and quinine for F656C-hERG was 771 ± 81 μM and 391 ± 40 μM, respectively. Quinine is more potent than quinidine in blocking F656C-hERG in comparison to blocking WT-hERG. Abbreviations: ctl, control; WT-hERG, wild type hERG; F656C-hERG, phenylalanine in 656 mutanted to cysteine.

**Figure 5:**
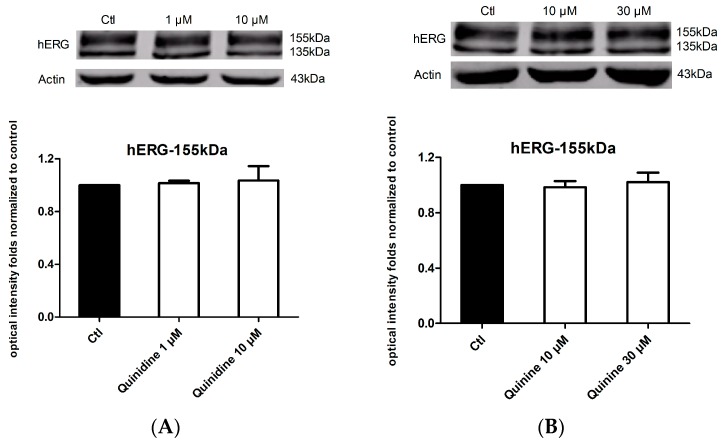
Effects of quinidine and quinine on hERG expression. (**A**) hERG expression levels in hERG-HEK cells under control conditions or 1 μM/10 μM quinidine treatment for 24 h. Mature hERG protein was not affected by 1 μM/10 μM quinidine. *n* = 5; (**B**) hERG expression levels in hERG-HEK cells under control conditions or 10 μM/30 μM quinine treatment for 24 h. Mature hERG protein was not affected by 10 μM/30 μM quinine. *n* = 5. Abbreviation: ctl, control.

To investigate the binding sites accounting for quinidine and quinine triggered hERG blockage, we tested the effects of the two drugs on a mutant hERG channel by injecting *Xenopus* oocytes with F656C-hERG cRNA. As shown in Figure 4A, quinidine 10 μM or quinine 100 μM remarkably decreased the WT-hERG current similar to the results in Figure 2A (quinidine is more potent than quinine). However, quinidine did not exert significant inhibition on F656C-hERG currents, and quinine 100 μM exhibited slight blockage of F656C-hERG. The mean extent of blockage of WT-hERG and F656C-hERG by quinidine and quinine at different concentrations is displayed in Figure 5B. Stereospecific blockage of WT exists, with quinidine being more potent than quinine. In contrast, this stereospecificity was reversed in the F656C mutation; the IC_50_ for quinidine and quinine was increased from 3.00 ± 0.03 μM and 44.0 ± 0.6 μM to 771 ± 81 μM and 391 ± 40 μM, respectively (quinine is more potent than quinidine in block of F656C-hERG). These data indicate that interactions with the F656 residue are responsible for the blockage of hERG by quinidine and quinine.

### 2.4. Effects of Quinidine and Quinine on the Expression of the hERG Channel

Considering the fact that up to 40% of hERG blockers presented combined acute blockage and trafficking defect [16], we next detect whether quinidine and quinine affect hERG expression by long-term treatment of hERG-HEK cells with the drugs for 24 h. As shown in Figure 4A, neither the mature 155 kDa hERG nor the immature 135 kDa hERG changed on western blots after incubation with 1 μM or 10 μM quinidine. Similarly, 10 μM or 30 μM quinine also did not change the expression level of the hERG channel (Figure 4B). Together, these results suggest that both quinidine and quinine do not affect the expression of the hERG protein.

### 2.5. Effects of Quinidine and Quinine on Drug-Induced hERG Trafficking Deficiency

**Figure 6:**
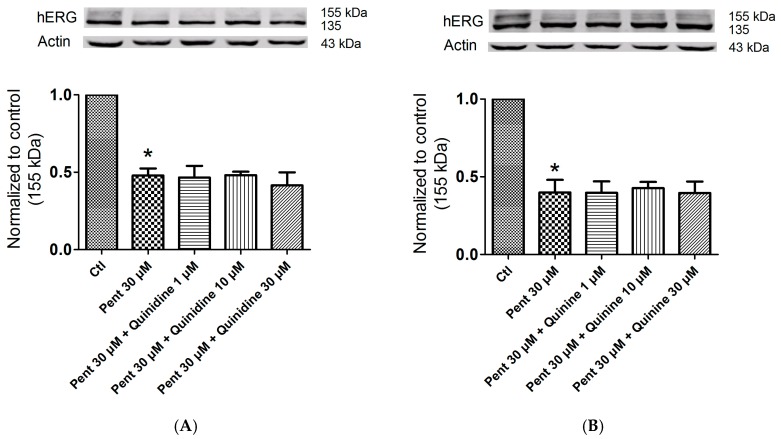

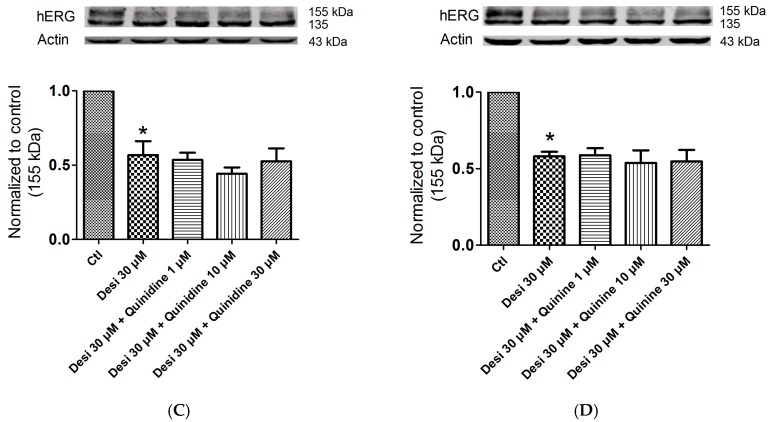
Effects of quinidine and quinine on the pentamidine- or desipramine-induced hERG defect. (**A**) Different concentrations of quinidine (1, 10, and 30 μM) did not upregulate the expression of 155-kDa hERG after 30 μM pentamidine treatment. * *p* < 0.05 vs. control, *n* = 3; (**B**) Different concentrations of quinine (1, 10, and 30 μM) did not upregulate the expression of 155-kDa hERG after 30 μM pentamidine treatment. * *p* < 0.05 vs. control, *n* = 3; (**C**) Different concentrations of quinidine (1, 10, and 30 μM) did not upregulate the expression of 155-kDa hERG after 30 μM desipramine treatment. * *p* < 0.05 vs. control, *n* = 3; (**D**) Different concentrations of quinine (1, 10, and 30 μM) did not upregulate the expression of 155-kDa hERG after 30 μM desipramine treatment. * *p* < 0.05 vs. control, *n* = 3. The hERG-HEK cells were incubated with the drugs for 24 h. Abbreviation: ctl, control.

A study reported that quinidine can be used as a pharmacological chaperone to rescue trafficking-deficient mutation hERG G601S based on its interaction with F656 of the hERG channel [17]. Hence, we suspect that quinidine or quinine could facilitate drug-induced hERG trafficking inhibition. Pentamidine and desipramine were reported to suppress hERG forward trafficking from the endoplasmic reticulum, and the aromatic amino F656 in S6 domain mediated the effects of pentamidine [16,19]. Moreover, desipramine produced acute blockage of the hERG channel at the binding sites of Y652 and F656 [20]. Here, we investigated whether quinidine and quinine could increase hERG forward trafficking restrained by pentamidine or desipramine. Unexpectedly, these two drugs showed no rescue effect on pentamidine or desipramine-reduced hERG protein. In Figure 6, pentamidine markedly reduced hERG expression on the membrane, but application of 1, 10, and 30 μM quinidine (A) or quinine (B) did not increase the 155-kDa hERG level in the presence of 30 μM pentamidine. A similar phenomenon was observed when quinidine (C) or quinine (D) was used in combination with desipramine. These data indicated that quinidine and quinine might have no rescue efficacy in drug-induced hERG inhibition.

## 3. Discussion

In the present study, we demonstrated that quinidine and quinine produce a stereoselective blockage of the hERG channel which is reflected in three aspects: quinine is ~14-fold less potent than quinidine; quinidine slows the deactivation of hERG, whereas quinine accelerates it; F656C mutation abrogates these stereoselective differences between quinidine and quinine. However, the hERG blockage by quinidine and quinine is not accompanied with a reduction in channel expression on the cell surface. In rescue experiments, both quinidine and quinine show no effect on the drug-induced hERG trafficking defect.

Quinidine and its diastereoisomer quinine share similar pharmacological activities in many aspects such as antiarrhythmia and antimalaria [21,22], whereas they display different clinical application profiles. Quinidine is widely used for atrial fibrillation and ventricular tachycardia [2]. Quinine is mainly used for malaria [3]. Although artemisinin is used as a substitute for other antimalarials as the first-line drug in the treatment of malaria, quinine and/or quinidine are still widely used for treating severe malaria because of the lack of availability of intravenous artesunate [3,23,24]. However, cardiotoxicity has become a major adverse effect during treatment with quinidine and quinine [4,5]. The cardiotoxicity of these drugs mainly focuses on delayed ventricular repolarization which increases the risk of LQTS and TdP. In addition, QT prolongation with quinine is about four times less than with quinidine [25]. Drug-induced LQTS is often associated with the inhibition of the hERG potassium channel [10]. Thus, we suspect that stereospecific blockage of hERG by quinidine and quinine contributes to their differed cardiotoxicity. Previous studies have reported stereospecific differences in the blockage of Kv1.5 by quinidine and quinine, but the mechanism was not illustrated [26]. In this study, stereospecific blockage of hERG by quinidine and quinine was observed, with quinidine being 14-fold more potent than quinine. Quinine at a concentration of 10 μM would be expected to have little effect (<20% block) on hERG, whereas a concentration of quinidine at 10 μM would be expected to produce 80% blockage of hERG. In patients, pharmacologically relevant concentrations of quinidine are in the range of 6–10 μM whereas for quinine they are in the range of 4–17 μM [21]. Since free concentrations of the two drugs are similar during the therapy of malaria, the difference in effects on the hERG channel may explain the moderate prolongation of QT intervals by quinine and its low propensity to proarrhythmia in humans. Next, we explored the mechanisms underlying this stereoselectivity. Among the binding sites that mediate drug-induced hERG blockage, the aromatic amino acid Y652 and F656 in segment S6 are two crucial determinants [27]. Recently, another aromatic amino acid F557 has been identified as a novel high affinity binding site for blockers, which is equally potent as Y652 [28]. The benzene ring of these amino acids is considered to interact with the benzene rings of various drugs through π bonds, thus producing blockage of hERG. Because of the influence of asymmetric carbon atoms, the benzene ring of quinidine and quinine are presented in different spatial orientations. Therefore, we hypothesized that the interaction with aromatic residue of hERG is involved in the stereospecific blockage of hERG by quinidine and quinine. In our research, we used site-directed mutagenesis to clarify the mechanism of that stereoselectivity. The results show that mutation of the F656C abrogates the stereoselective blocking by quinidine and quinine, and decreases the affinity for quinidine by approximately 250-fold (Figure 4). For F656C-hERG, quinidine can still block the channel but only at high and suprapharmacologic concentrations (771 ± 81 μM). These data indicate that F656 is a high affinity binding site for quinindine or quinine, whereas there appears to be another lower affinity binding site elsewhere. Previous radioligand binding studies confirm the presence of high and low affinity binding sites for ^3^H-dofetilide to cardiac myocytes [29].

Considering the fact that up to 40% of hERG blockers presented combined acute blockage and trafficking defect [16], we tested the effect of quinidine and quinine on hERG expression, yet neither drug had an effect (Figure 5). Even so, it has been reported that quinidine increased hERG expression of the trafficking-deficient pore mutation G601S-hERG [17]. High-affinity hERG channel–blocking drugs E4031, astemizole, cisapride and quinidine all produced reverse effects, and the rescue was dependent on their interaction with F656. Successful pharmacological rescue also occurred when E4031, astemizole, terfenadine and fexodenadine were used for the rescue of N470D-hERG, another trafficking-deficient mutation [18,30]. It is possible that quinidine could correct the trafficking of N470D-hERG, similar to astemizole. Regarding the drug-induced hERG trafficking defect, astemizole presented a rescue efficacy with respect to the pentamidine- and berberine- induced hERG defect [19,31], which was verified in our research. Considering that the F656 residue contributes to the stereoselective difference between quinidine and quinine in hERG blockage, we formulated the hypothesis that the two drugs can produce a stereoselective rescue effect on the drug-triggered hERG trafficking defect. Pentamidine and desipramine were reported to suppress hERG forward trafficking to the cell surface, and the aromatic amino F656 mediated the effects of pentamidine [16,19]. Moreover, desipramine produced acute blockage of the hERG channel at the binding sites of Y652 and F656 [20]. Beyond our expectation, both quinidine and quinine presented no rescue ability for the drug-triggered hERG defect (Figure 6).

## 4. Materials and Methods

### 4.1. Isolation and Maintenance of Xenopus Oocytes

Female *Xenopus laevis* were placed on ice after anaesthetization by 0.2% tricaine (Sigma-Aldrich Co., St Louis, MO, USA) for 15 min. They were dissected and oocytes clusters were removed and transferred to ND96 solution (96 mM NaCl, 2 mM KCl, 1 mM MgCl_2_, 1.8 mM CaCl_2_ and 5 mM HEPES, pH 7.5). Then, the cell clusters were digested with 1 mg·mL^−1^ Type II collagenase (Sigma-Aldrich Co., St. Louis, MO, USA) in a Ca^2+^-free ND96 solution. The isolated oocytes were incubated at 18 °C in ND96 solution supplemented with penicillin-streptomycin solution (Beyotime, Beijing, China).

### 4.2. Site-Directed Mutagenesis and cRNA Injection

The cDNA expression construct in the pSP64 transcription vector (Promega, Madison, WI, USA) was conducted as described previously [32]. Mutation constructs were confirmed by restriction enzyme and DNA sequence analyses. cRNA for injection into oocytes was prepared with the mMESSAGE mMACHINE kit (Ambion, Dallas, TX, USA) using SP6 RNA polymerase after linearization of the cDNA with *EcoR1*, according to manufacturer’s protocols. cRNAs were dissolved in diethylpyrocarbinate-treated sterile water, stored at −80 °C, and diluted immediately prior to injection. Stage V and VI oocytes were injected with 10 nL of cRNA encoding wild-type (WT) hERG or F656C-hERG.

### 4.3. Electrophysiology in Xenopus Oocytes

Whole cell currents were recorded 2–5 days after cRNA injection using a GeneClamp 500 amplifier, a Pentium Computer with a Digidata 1200 computer interface (Axon Instruments, Sunnyvale, CA, USA), and standard two-electrode voltage clamp techniques. Electrodes were filled with 3 M KCl with resistance of approximately 0.8–1.2 MΩ when measured in the modified frog Ringers solution (114 mM NaCl, 2.5 mM KCl, 1 mM MgCl_2_, 1.8 mM CaCl_2_, and 10 mM HEPES, pH 7.2). P-clamp software (Axon Instruments) was used to generate voltage-clamp commands, acquire membrane currents, and analyze digitized data. For the steady-state IC_50_ measurements, the oocytes were pulsed continuously from a holding potential of −80 to 0 mV for 2.5 s with an interpulse interval to −60 mV for 1.5 s during the entire 20 min superfusion with drug. Data were fit to a monoexponential function. Experiments were conducted at room temperature (22 °C).

Quinidine and quinine (Sigma Chemical, St. Louis, MO, USA) were dissolved in the external solution to obtain the desired concentrations.

### 4.4. Expression of hERG in Ltk^−^ Cells

*Ltk^−^* cells were cultured in Dulbecco’s Modified Eagle’s Medium (DMEM; Hyclone, Logan, UT, USA) supplemented with 10% horse serum. The cells were transfected with WT-hERG cDNA using Lipofectamine 2000 (Invitrogen, Carlsbad, CA, USA). In brief, cells were plated in 25-mm dishes at the required density and incubated overnight. Then, 3–4 h prior to transfection, the media in the dishes was replaced with 4.5 mL DMEM. Next, 250 μL opti-MEM Reduced-Serum Medium (GIBCO, Grand Island, NY, USA) and 12.5 μL Lipofectamine 2000 (Invitrogen, Carlsbad, CA, USA) were added to a tube labeled A, 250 μL opti-DMEM and 4 μg cDNA were added to a tube labeled B, and then solution A and solution B were mixed. After incubation at room temperature for 20 min, the mixture was added to cells in the dishes. Green fluorescent protein (GFP) was coexpressed to assess the transfection efficiency. Transfected *Ltk^−^* cells were cultured in DMEM supplemented with 10% horse serum and 0.25 mg/mL G418 (GIBCO, Grand Island, NY, USA). Recordings were made 48 h after transfection.

### 4.5. Electrophysiology in Ltk^−^ Cells

A whole-cell voltage clamp was used to record currents in transfected *Ltk^−^* cells. Cells were superfused with a HEPES-buffered Tyrode’s solution (140 mM NaCl, 4 mM KCl, 1 mM MgCl_2_, 1 mM CaCl_2_, 5.5 mM Glucose, and 10 mM HEPES with pH 7.4) at room temperature (22 °C). Electrodes were filled with an intracellular pipette filling solution (110 mM potassium aspartate, 4 mM MgCl_2_, 4.2 mM K_2_ATP, 1 mM CaCl_2_, 8 mM NaCl, 5 mM HEPES and 10 mM EGTA, pH 7.2) with tip resistances of 1 to 4 MΩ. The hERG currents were elicited from a holding potential of −80 mV by a depolarizing pulse to 0 mV for 4 s, followed by a second pulse to −60 mV for 1 s.

### 4.6. Western Blot Analysis

hERG-HEK293 cells, stably expressing the wild-type hERG gene, were used to perform the Western blot experiments. The cells were cultured at 37 °C under 5% CO_2_ in DMEM (Hyclone, Logan, UT, USA) supplemented with 10% fetal bovine serum (FBS; Bioind, Cromwell, CT, USA). The cells expressing the hERG protein were obtained by using G418 (400 μg·mL^−1^; Invitrogen, Carlsbad, CA, USA) selection. The Western blot procedure was described previously [31]. In brief, lysed cells were harvested after addition of RIPA buffer (Beyotime, China) containing 1% phenylmethanesulfonyl fluoride (PMSF; Beyotime, Beijing, China). Protein concentration was determined using NanoDrop 2000C (Thermo Fisher Scientific, Wyman, MA, USA). The proteins (100 μg·per sample) were separated using 8% sodium dodecyl sulfate polyacrylamide gel electrophoresis (SDS-PAGE) and transferred to a polyvinylidene fluoride (PVDF) membrane. Subsequently, the membrane was probed with primary antibodies against hERG (Santa Cruz, Santa cruz, CA, USA) or actin (Zhongbin Jinqiao, Beijing, China) overnight. After incubation with horseradish peroxidase–conjugated secondary antibodies (Li-CoR, Lincoln, NE, USA), the membranes were examined for bands using the Odyssey Instrument (Li-CoR, Lincoln, NE, USA). The blots were analyzed and quantified with Image Studio software. Quinidine and quinine were dissolved in ethanol as a stock (10 μM) and were diluted in DMEM before use. Cells were incubated with the drugs for 24 h at 37 °C.

### 4.7. Data Analysis

The data are expressed as the means ± standard error of the mean (S.E.M). Differences between the means were obtained using analysis of variance (ANOVA) and Student’s *t* test. *p* values <0.05 were considered statistically significant.

## 5. Conclusions

In conclusion, stereoselective differences in the configuration of quinidine and quinine mediate differences in affinity of these molecules for interaction with the F656 residue of hERG. Our study focuses on the comprehensive effects of quinidine and quinine on the hERG channel, including acute blockage, channel protein expression and rescue efficacy with respect to the drug-induced channel trafficking defect, which provides useful information for the basic research on drug-induced LQTS.

## References

[1] Weinreb S.M. (2001). Chemistry. Synthetic lessons from quinine. Nature.

[2] Salerno D.M. (1992). Quinidine: Is it a good drug or a bad drug?. Postgrad. Med..

[3] Achan J., Talisuna A.O., Erhart A., Yeka A., Tibenderana J.K., Baliraine F.N., Rosenthal P.J., D’Alessandro U. (2011). Quinine, an old anti-malarial drug in a modern world: Role in the treatment of malaria. Malar. J..

[4] Bonington A., Davidson R.N., Winstanley P.A., Pasvol G. (1996). Fatal quinine cardiotoxicity in the treatment of falciparum malaria. Trans. R. Soc. Trop. Med. Hyg..

[5] Wroblewski H.A., Kovacs R.J., Kingery J.R., Overholser B.R., Tisdale J.E. (2012). High risk of QT interval prolongation and torsades de pointes associated with intravenous quinidine used for treatment of resistant malaria or babesiosis. Antimicrob. Agents Chemother..

[6] Mirro M.J., Watanabe A.M., Bailey J.C. (1981). Electrophysiological effects of the optical isomers of disopyramide and quinidine in the dog. Dependence on stereochemistry. Circ. Res..

[7] Sheldon R.S., Rahmberg M., Duff H.J. (1990). Quinidine/quinine: Stereospecific electrophysiologic and antiarrhythmic effects in a canine model of ventricular tachycardia. J. Cardiovasc. Pharmacol..

[8] Kinoshita A., Yamada H., Kotaki H., Kimura M. (2010). Effects of anti-malarial drugs on the electrocardiographic QT interval modelled in the isolated perfused guinea pig heart system. Malar. J..

[9] Touze J.E., Heno P., Fourcade L., Deharo J.C., Thomas G., Bohan S., Paule P., Riviere P., Kouassi E., Buguet A. (2002). The effects of antimalarial drugs on ventricular repolarization. Am. J. Trop. Med. Hyg..

[10] Yang T., Snyders D., Roden D.M. (2001). Drug block of I Kr: Model systems and relevance to human arrhythmias. J. Cardiovasc. Pharmacol..

[11] Sanchez-Chapula J.A., Ferrer T., Navarro-Polanco R.A., Sanguinetti M.C. (2003). Voltage-dependent profile of human *Ether-a-go-go*-related gene channel block is influenced by a single residue in the S6 transmembrane domain. Mol. Pharmacol..

[12] Villoutreix B.O., Taboureau O. (2015). Computational investigations of herg channel blockers: New insights and current predictive models. Adv. Drug Deliv. Rev..

[13] Vonderlin N., Fischer F., Zitron E., Seyler C., Scherer D., Thomas D., Katus H.A., Scholz E.P. (2015). Anesthetic drug midazolam inhibits cardiac human *ether-a-go-go*-related gene channels: Mode of action. Drug Des. Dev. Ther..

[14] Zhang K., Zhi D., Huang T., Gong Y., Yan M., Liu C., Wei T., Dong Z., Li B., Yang B. (2014). Berberine induces herg channel deficiency through trafficking inhibition. Cell. Physiol. Biochem..

[15] Shi Y.Q., Yan C.C., Zhang X., Yan M., Liu L.R., Geng H.Z., Lv L., Li B.X. (2015). Mechanisms underlying probucol-induced HERG-channel deficiency. Drug Des. Dev. Ther..

[16] Dennis A.T., Nassal D., Deschenes I., Thomas D., Ficker E. (2011). Antidepressant-induced ubiquitination and degradation of the cardiac potassium channel herg. J. Biol. Chem..

[17] Ficker E., Obejero-Paz C.A., Zhao S., Brown A.M. (2002). The binding site for channel blockers that rescue misprocessed human long qt syndrome type 2 *ether-a-gogo*-related gene (HERG) mutations. J. Biol. Chem..

[18] Rajamani S., Anderson C.L., Anson B.D., January C.T. (2002). Pharmacological rescue of human K^+^ channel long-QT2 mutations: Human *ether-a-go-go*-related gene rescue without block. Circulation.

[19] Dennis A.T., Wang L., Wan H., Nassal D., Deschenes I., Ficker E. (2012). Molecular determinants of pentamidine-induced herg trafficking inhibition. Mol. Pharmacol..

[20] Hong H.K., Park M.H., Lee B.H., Jo S.H. (2010). Block of the human *ether-a-go-go*-related gene (HERG) K^+^ channel by the antidepressant desipramine. Biochem. Biophys. Res. Commun..

[21] Sheldon R., Duff H., Koshman M.L. (1995). Antiarrhythmic activity of quinine in humans. Circulation.

[22] White N.J. (2007). Cardiotoxicity of antimalarial drugs. Lancet Infect. Dis..

[23] Kong L.Y., Tan R.X. (2015). Artemisinin, a miracle of traditional chinese medicine. Nat. Prod. Rep..

[24] Sanders N.G., Meyers D.J., Sullivan D.J. (2014). Antimalarial efficacy of hydroxyethylapoquinine (SN-119) and its derivatives. Antimicrob. Agents Chemother..

[25] White N.J., Looareesuwan S., Warrell D.A. (1983). Quinine and quinidine: A comparison of EKG effects during the treatment of malaria. J. Cardiovasc. Pharmacol..

[26] Snyders D.J., Yeola S.W. (1995). Determinants of antiarrhythmic drug action. Electrostatic and hydrophobic components of block of the human cardiac HKV1.5 channel. Circ. Res..

[27] Mitcheson J.S., Chen J., Lin M., Culberson C., Sanguinetti M.C. (2000). A structural basis for drug-induced long qt syndrome. Proc. Natl. Acad. Sci. USA.

[28] Saxena P., Zangerl-Plessl E.M., Linder T., Windisch A., Hohaus A., Timin E., Hering S., Stary-Weinzinger A. (2016). New potential binding determinant for herg channel inhibitors. Sci. Rep..

[29] Duff H.J., Feng Z.P., Sheldon R.S. (1995). High- and low-affinity sites for [3H]dofetilide binding to guinea pig myocytes. Circ. Res..

[30] Zhou Z., Gong Q., January C.T. (1999). Correction of defective protein trafficking of a mutant HERG potassium channel in human long QT syndrome. Pharmacological and temperature effects. J. Biol. Chem..

[31] Yan M., Zhang K., Shi Y., Feng L., Lv L., Li B. (2015). Mechanism and pharmacological rescue of berberine-induced HERG channel deficiency. Drug Des. Dev. Ther..

[32] Sanguinetti M.C., Xu Q.P. (1999). Mutations of the S4–S5 linker alter activation properties of HERG potassium channels expressed in *Xenopus* oocytes. J. Physiol..

